# Acute Renal Failure Due to Amyloidosis Associated With Intravenous Heroin Use

**DOI:** 10.7759/cureus.88860

**Published:** 2025-07-27

**Authors:** Ramya Malchira, Michael Shye, Raghu Konanur, Harpreet Sidhu, Jonathan Zuckerman

**Affiliations:** 1 Nephrology, David Geffen School of Medicine, University of California, Los Angeles, Los Angeles, USA; 2 Medicine/Nephrology, David Geffen School of Medicine, University of California, Los Angeles, Los Angeles, USA; 3 Pathology and Laboratory Medicine, University of California, Los Angeles, Los Angeles, USA

**Keywords:** aa amyloidosis, acute kidney injury, adult nephrotic syndrome, heroin use, intravenous drug use (ivdu), nephrotic syndrome, skin popping

## Abstract

Amyloidosis encompasses a group of disorders characterized by the accumulation of insoluble protein fibrils within the extracellular matrix. These fibrils originate from low molecular weight protein subunits, many of which circulate naturally in the bloodstream. The resulting deposits can occur in nearly any organ, with the clinical picture shaped by the type, distribution, and extent of amyloid involvement. Classification of amyloidosis is based on the identity of the precursor protein. Amyloid A (AA) amyloidosis, a systemic condition arising from chronic inflammation, is less common and most often linked to persistent inflammatory states, including chronic intravenous drug use.

This case report describes a 54-year-old male with a history of injection heroin use who presented to the emergency department with a six-month history of progressive fatigue, exertional shortness of breath, and foamy urine. Laboratory evaluation revealed acute kidney injury (AKI), nephrotic range proteinuria, and significant anemia. A kidney biopsy confirmed the presence of AA-type amyloid deposits. AA amyloidosis is frequently observed in patients with autoimmune or chronic inflammatory conditions and has also been reported in individuals who engage in chronic intravenous and subcutaneous heroin use, known colloquially as "skin popping." Patients with subcutaneous heroin use carry a heightened risk of secondary amyloidosis, particularly affecting kidney function.

Clinicians should maintain a high index of suspicion for AA amyloidosis in patients with a history of long-term IV drug use who present with proteinuria and AKI.

## Introduction

Chronic IV heroin use is associated with several medical conditions, including renal abnormalities such as acute kidney injury (AKI), rhabdomyolysis, and nephrotic syndrome [1,2]. Heroin use, intravenous and subcutaneous, which is also known as “skin popping,” has been associated with amyloid A (AA) amyloidosis [2]. AA amyloidosis (previously known as secondary amyloidosis) is a rare disorder marked by the deposition of fibrils composed of fragments or intact serum amyloid A (SAA) protein in extracellular tissues. SAA is an acute-phase reactant produced by the liver. The activation of macrophages can trigger the release of pro-inflammatory cytokines, including interleukin-1 (IL-1) and interleukin-6 (IL-6), which promote the production of SAA [3,4]. Within circulating macrophages, the precursor protein undergoes cleavage, producing smaller AA protein fragments that subsequently accumulate in various tissues. AA amyloidosis may develop secondary to long-standing inflammatory disorders, including autoimmune diseases like rheumatoid arthritis, juvenile idiopathic arthritis, and ankylosing spondylitis, as well as conditions marked by persistent inflammation, such as inflammatory bowel disease, familial periodic fever syndrome, chronic infectious diseases, and subcutaneous and IV drug use.

One of the postulated mechanisms in the pathogenesis of amyloidosis in this patient population is chronic immunological stimulation by exogenous antigens or multiple acute inflammatory episodes [5-7].

## Case presentation

We report a case of a 54-year-old man with a history of polysubstance abuse, chronic hepatitis C infection, and hypertension, who presented to the emergency room with a six-month history of progressive generalized fatigue, dyspnea on exertion, and foamy urine. He was diagnosed with hypertension a year prior to this hospitalization and had self-discontinued anti-hypertensive medications. His history was notable for several years of illicit drug use, including IV heroin, cocaine, and amphetamines. He had last used IV heroin two days prior to presentation. Vital signs were notable for elevated blood pressure of 173/95 mm Hg. Physical examination was notable for conjunctival pallor and bilateral lower extremity 1+ pitting edema below the knees. Cardiopulmonary examination was unremarkable.

Initial laboratory results were significant for AKI and nephrotic syndrome, as displayed in Table 1. Serum and urine immunofixation revealed monoclonal free lambda light chain, as displayed in Table 1. Pertinent negative laboratory tests included anti-nuclear antibody (ANA), anti-neutrophil cytoplasmic antibodies (ANCA), and complement C3 and C4.

**Table 1:** Notable laboratory findings. IgA: immunoglobulin A; Hepatitis C RNA PCR: hepatitis C RNA polymerase chain reaction.

Pertinent lab data	Results	Reference range
Serum creatinine	6.1	0.7-1.2 mg/dL
Urea nitrogen	60	6-20 mg/dL
Total CO2	14	22-29 mmol/L
Serum albumin	2.4	3.5-5.2g/dL
Urine protein-to-creatinine ratio	14949	22-128 mg/g creatinine
Hemoglobin	8.0	14-18 g/dL
IgA, serum	1860	726-1521 mg/dL
Kappa light chain, free	76.1	0.33-1.94 mg/dL
Lambda light chain, free	22.80	0.57-2.63 mg/dL
Kappa/lambda, free	3.34	0.26-1.65
Hepatitis C RNA PCR	32,200 IU/mL	Not detected

Kidney ultrasound showed normal kidney sizes, loss of cortico-medullary differentiation, and no hydronephrosis.

**Figure 1:**
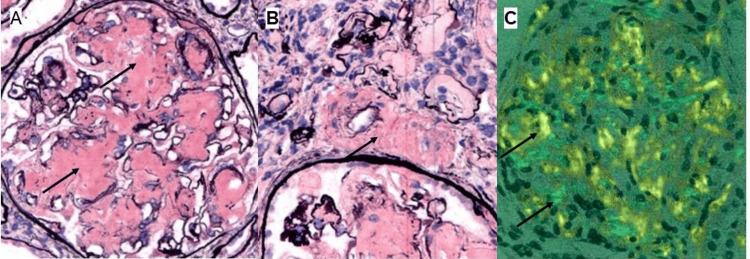
Kidney biopsy findings. (A) Light micrographs of Jones' silver-stained sections demonstrate extensive glomerular infiltration by acellular amorphous silver-negative material (arrows), which expands mesangial spaces and capillary walls. (B) Similar material is also demonstrated in arteriolar walls (arrows) and was present throughout interstitial spaces. (C) Congo red-stained sections demonstrate anomalous colors (arrows) under polarized light. All original magnifications at 400x.

A kidney biopsy was performed (Figure 1), which showed amyloidosis with extensive glomerular, interstitial, and arteriolar involvement. Congo red staining was confirmatory, which demonstrated anomalous colors under polarized light. The specimen was further evaluated by mass spectrometry, which confirmed type AA amyloidosis.

## Discussion

Amyloidoses are a group of disorders characterized by the deposition of highly ordered fibrils in extracellular tissues, which eventually leads to organ failure and death. Amyloidosis is classified based on the precursor protein involved and is further categorized as either wild-type (acquired) or mutant (associated with germline pathogenic variants). To date, 42 proteins have been identified as capable of forming amyloid fibrils in humans [7,8]. A definitive diagnosis requires tissue biopsy, where amyloid deposits exhibit characteristic gross pathologic and microscopic appearance, demonstrating characteristic anomalous colors with polarized light microscopy of Congo red-stained tissue [9,10]. Determining the amyloid type is critical for guiding prognosis, treatment, and genetic counseling.

Among systemic amyloidoses, the most frequently encountered subtypes are light chain (AL) amyloidosis and transthyretin (ATTR) amyloidosis, representing approximately 56% and 21% of cases, respectively. In a single-center cohort, AA amyloidosis accounted for around 8% of cases [7,10]. SAA, an apolipoprotein primarily synthesized by hepatocytes and also produced by macrophages, endothelial cells, and smooth muscle cells, is the precursor protein in AA amyloidosis. As an acute-phase reactant, SAA is involved in processes such as joint destruction in inflammatory arthritis and tumor metastasis [7]. Chronic elevation of SAA, driven by inflammatory cytokines such as tumor necrosis factor α (TNFα), interleukin-1β (IL-1β), and interleukin-6 (IL-6) in underlying persistent infection, autoimmune disease, or chronic substance use, can lead to AA amyloid deposition. Patients often exhibit elevated markers of inflammation, such as erythrocyte sedimentation rate (ESR) and C-reactive protein (CRP); however, SAA level remains the most specific marker [11,12]. SAA levels correlate with renal progression and amyloid load by serum amyloid P (SAP) scintigraphy [13]. Currently, the SAA level is only commercially available in Europe [7].

AA amyloidosis develops in about 5% of patients with sustained elevation of SAA. The kidney is often the main target, which is affected universally in all patients with AA amyloidosis [6,7]. Proteinuria and a decrease in renal function are common presenting features. In roughly 5% of cases, proteinuria is absent. When amyloid primarily affects the renal vasculature or tubules, patients may develop significant kidney dysfunction with comparatively modest levels of proteinuria. Kidney involvement is followed by gastrointestinal disturbances such as diarrhea, constipation, malabsorption, and hepatosplenomegaly. In contrast to amyloid light chain (AL) amyloidosis, congestive heart failure, peripheral neuropathy, macroglossia, and carpal tunnel syndrome occur infrequently. Kidney biopsy is generally required for a definitive diagnosis of renal amyloidosis [12].

Heroin use has increased significantly across most demographic groups in the United States of America, with rates remaining highest among males, persons aged 18-25 years, those living in urban areas, and persons with no health insurance or with Medicaid [14]. Recent epidemiological data suggest that persistent inflammation associated with heroin use is becoming an increasingly common underlying cause of AA amyloidosis [5]. In a large cohort of 38 patients with AA amyloidosis described by Sharma et al. [15], 95% had a documented history of heroin use. Heroin use was associated with an approximately 170-fold increased risk of developing AA amyloidosis compared to individuals without a history of heroin use. The authors demonstrated that AA amyloidosis was more commonly observed in White individuals, particularly those using a form of heroin known as “black tar heroin,” and was frequently associated with underlying hepatitis C infection. Histopathological analysis showed glomerular involvement in all cases, with vascular and tubulointerstitial involvement also occurring commonly. Progression to end-stage kidney disease (ESKD) in most patients was within a short time, with a median time to ESKD of 2.4 years. The authors also noted a higher mortality in heroin-using patients who developed AA amyloidosis.

Specific therapy of renal amyloidosis is guided by the type of amyloid deposition. Treatment of AA amyloidosis is targeted at treating the underlying process driving the production of SAA. In a review by Lachmann et al. of 374 patients with AA amyloidosis who were followed for a median of 86 months, the median survival from diagnosis was 133 months. Some of the poor prognostic features include advanced age, hypoalbuminemia, ESKD at diagnosis, and higher SAA levels during follow-up [12]. Those patients who progress to ESKD can be treated with either dialysis or renal transplantation. Renal amyloidosis should be an important differential diagnosis to consider when heroin users present to the hospital with proteinuria and AKI. Early diagnosis and prompt control of the underlying inflammatory condition are critical to prevent irreversible kidney damage and improve survival outcomes in patients with AA amyloidosis [6]. Our patient underwent hemodialysis for three years following diagnosis and ultimately succumbed to related complications.

## Conclusions

AA amyloidosis is a recognized complication of chronic inflammatory disorders. Heroin use, whether intravenous or subcutaneous (“skin popping”), is frequently associated with the development of renal AA amyloidosis. This diagnosis should be strongly considered in patients presenting with proteinuria and renal insufficiency. Timely diagnosis, confirmed by kidney biopsy, is critical to facilitate early intervention and prevent further progression of kidney damage.
